# Efficacy and Safety of Moxibustion for Postherpetic Neuralgia: A Systematic Review and Meta-Analysis

**DOI:** 10.3389/fneur.2021.676525

**Published:** 2021-08-26

**Authors:** Qiqi Wu, Hantong Hu, Dexiong Han, Hong Gao

**Affiliations:** ^1^The Third Clinical College of Zhejiang Chinese Medical University, Hangzhou, China; ^2^Department of Acupuncture and Moxibustion, The Third Affiliated Hospital of Zhejiang Chinese Medical University, Hangzhou, China

**Keywords:** postherpetic neuralgia, moxibustion, systematic reveiw, herpes zoster, meta-analysis

## Abstract

**Background:** Postherpetic neuralgia (PHN) is one of the most common complications of herpes zoster (HZ), and there is still a lack of effective therapies. An increasing number of studies have found that compared to traditional therapy, moxibustion treatment is beneficial for the treatment of PHN, although current evidence remains inconclusive. This systematic review and meta-analysis of randomized controlled trials (RCTs) aimed to evaluate the efficacy and safety of moxibustion for PHN.

**Methods:** We conducted a broad literature review of a range of databases from inception to December 2020, including the Cochrane Library, PubMed, EMBASE, Web of Science, Clinical Trails, China National Knowledge Infrastructure (CNKI), VIP Database for Chinese Technical Periodicals (VIP), China Biomedical Network Information, and Wanfang databases. We included RCTs that compared moxibustion to pharmacological therapies, herbal medicine, or no treatment for treating PHN. The main outcome measure was efficacy rate and Visual Analog Scale (VAS); the secondary outcome measure was adverse events. Data accumulation and synthesis included meta-analysis, publication bias, sensitivity analysis, risk-of-bias assessment, and adverse events.

**Results:** We included 13 RCTs involving 798 patients. Compared with the controls (pharmacological therapies, herbal medicine, or no treatment), moxibustion achieved a significantly higher efficacy rate (odds ratio [OR]: 3.65; 95% [confidence interval]: [2.32, 5.72]; *P* < 0.00001). Subgroup analysis of the distinct moxibustion modalities showed that both Zhuang medicine medicated thread and thunder-fire moxibustions obtained higher clinical efficacy than the control group. Compared with the controls, moxibustion resulted in significantly lower scores on the VAS (Weighted Mean Difference (MD) = −1.79; 95% CI: [−2.26, −1.33]; *P* < 0.00001). However, there was no significant difference in terms of safety between moxibustion and the controls (OR = 0.33; 95% CI [0.06, 1.77]; *P* = 0.19).

**Conclusion:** Due to the lack of methodological quality as well as the significant heterogeneity of the included studies, it remains difficult to draw a firm conclusion on the efficacy and safety of moxibustion for the treatment of PHN. Future high-quality studies are urgently needed.

## Introduction

Herpes zoster (HZ) is an acute painful blister rash caused by the reactivation of the dormant varicella-zoster virus in the sensory ganglion (1–5). Postherpetic neuralgia (PHN) is characterized by neuropathic pain caused by HZ, which can persist after at least 1–6 months from the HZ rash onset.

The pathogenesis of postherpetic neuralgia is complicated and is related to abnormal sympathetic nerve function, inflammatory reactions, reduced levels of immune factors, and central sensitization (6). Some researchers believe that nerve injury induced by herpes zoster leads to increased excitability of the spinal cord neurons and peripheral transmission fibers, increased spontaneous and induced neuronal sensitization, increased spontaneous discharge without stimulation, and an increased nociceptive stimulation response to neuropathic pain caused by the activation of the descending facilitation system after injury, including the gray matter, anterior cingulate gyrus, and hypothalamus (7).

The incidence of herpes zoster in China is ~7.7%, the incidence of PHN is ~2.3%, and the probability of HZ patients developing PHN is 29.8%; this incidence increases significantly with age (8). The prevalence of PHN increases by up to 70% with age, and the incidence of PHN is higher in females than in males (3). The incidence of PHN is not only related to age and gender but also the degree of pain during the onset of infection by herpes zoster, the duration of shingles, the location of zona involvement, and whether or not an autoimmune disease is involved (9).

PHN is a chronic and persistent pain. As a typical form of neuropathic pain, patients with PHN often have hyperalgesia, spontaneous pain, sensory loss, abnormal mechanical pain, and other clinical symptoms; collectively, these symptoms can have a severe effect on the physical and mental health of patients as they are often unable to dress and walk (10, 11). Clinical manifestations include persistent burning pain, shooting, stabbing, and electric shock tactile pain. The condition is prolonged, and the pain can be severe, persistent, and often unbearable. In some patients, the pain cannot be effectively controlled for a period of more than 3 years. Patients with severe pain over long time periods suffer greatly and are often depressed. This can seriously affect the quality of life and daily work of such patients, leading to insomnia, anxiety, depression, and even suicide (12, 13). With its high incidence and persistent and severe pain, PHN can also exert serious effects on the lives of elderly patients.

Presently, the first-line treatments for PHN include serotonin and noradrenaline reuptake inhibitors, tricyclic antidepressants (e.g., amitriptyline and nortriptyline), opioids (e.g., oxycodone) (14–16), antiepileptic drugs (e.g., gabapentin and pregabalin), and topical medications (e.g., capsaicin and lidocaine) (14, 16). OnabotulinumtoxinA therapy (17, 18) is also effective for PHN, which has recently gained support from a growing body of evidence. Nevertheless, only half of the patients who take these drugs report satisfactory results (12), and a considerable number of patients report that their level of pain does not improve following these treatments. Furthermore, these treatments are associated with many adverse reactions and a high recurrence rate (19–21).

Moxibustion is an important aspect of traditional Chinese medicine (TCM). The combination of the heat provided by moxibustion and herbs (moxa) can help to prevent disease and can exert notable therapeutic effects (22). In recent years, moxibustion has been widely investigated due to its safety, simplicity, and efficacy. A number of clinical trials have investigated the use of moxibustion for treating PHN. Nevertheless, evidence regarding the efficacy of moxibustion for the treatment of PHN remains inconclusive. Therefore, we conducted a meta-analysis of randomized controlled trials (RCTs) to evaluate the efficacy of moxibustion for PHN and aimed to provide a reliable clinical basis for the use of moxibustion in the treatment of PHN.

## Methods

This study, a systematic review and meta-analysis, is reported in accordance with the Preferred Reporting Items for Systematic Reviews and Meta-Analyses (PRISMA) guidelines (23), as shown in Appendix S1. The protocol was registered in PROSPERO with the identification number CRD42021233361.

### Database and Search Strategy

Two authors (QQW and HTH) searched several databases from inception to December 2020, including The Cochrane Library, PubMed, Chinese National Knowledge Infrastructure (CNKI), EMBASE, VIP Database for Chinese Technical Periodicals (VIP), Web of Science, Clinicaltrails.gov, Wanfang Data Information Site, and the China Biomedical Network Information Database. Research articles were limited to those written in either Chinese or English.

The following search terms were used for PubMed: #1: “postherpetic neuralgia” OR “herpes zoster” OR “zoster herpes” OR “zona” OR “zoster” OR “shingles” (in the title or the text of the abstract); #2: “moxibustion” OR “moxabustion” OR “moxa” (in the title or the text of the abstract); #3: “randomized controlled trial” OR “controlled clinical trial” OR “randomized clinical trial” OR “clinical trial” (in the title or the text of the abstract). #1 AND #2 AND #3. The search strategy for each database was provided in Appendix S2.

### Inclusion Criteria

The study types involved RCTs. The participants were patients diagnosed with PHN and where the duration of the disease is at least 1 month. Interventions included moxibustion, either alone or in combination with the same active treatment(s) as the control group. Controls included active treatments (e.g., pharmacological therapies and herbal medicine) or no treatment.

### Exclusion Criteria

Publications were excluded if the experimental group involved acupuncture intervention. We also excluded articles that were duplicated studies, animal experiments, master's theses and conference articles, and studies with missing data for extraction. In addition, we excluded trials with samples from “special age groups,” meaning trials which only involved PHN patients with a relatively small age range (e.g., elderly patients), because the baseline of the age of such a study would be very heterogeneous when compared with other included studies.

### Outcome Measures

The main outcomes could include either or both of these as follows.

In terms of *Clinical Efficacy Rate*, according to the Criteria of Diagnosis and Therapeutic Effects for TCM Disease and Syndrome (CDTETCMDS) (24), this is defined as (1) the disappearance of physical signs and pain in the rash distribution area (significantly improved); (2) when pain in the primary rash distribution area was obviously alleviated (improved); and (3) after treatment, if the pain was not improved (ineffective) (24). Subsequently, patients who were classified as “significantly improved” or “improved” were considered as patients showing pain improvement while those classified as “ineffective” were considered as patients without pain improvement. “Effective” is defined as the total number of patients classified as “significantly improved” or “improved,” by which the clinical efficacy rate was calculated. Other evaluation criteria of clinical efficacy rate with a comparable definition were also considered.

*Visual Analog Scale (VAS)*. A 10 cm ruler was used for patients to grade their pain symptoms (0–10 points, 1 point per 1 cm). Grade 0 represented no pain while grade 10 represented unbearable pain.

Secondary outcomes were defined as adverse events associated with moxibustion.

### Study Selection

Two independent authors (QQW and HTH) reviewed the titles and abstracts of published articles to identify appropriate RCTs. Only recent information was included when datasets overlapped or duplicated data. Then, the authors searched the entire text of selected articles for related research that was not identified in the literature searches. The same two independent authors (QQW and HTH) then reviewed the entire text of the identified articles to screen those that satisfied the inclusion criteria. Differences between the two authors were resolved through discussion and consensus with a third author (DXH).

### Data Extraction and Management

Two independent authors (QQW and HTH) retrieved information from the included RCTs, including the specifics of the research, populations, interventions, and data measurements. A third author (DXH) resolved differences related to data extraction through conversation and consensus.

### Evaluating the Quality of the Evidence Provided by the Included Studies

We evaluated the quality of the publications included in this meta-analysis by referring to The Cochrane Handbook for Systematic Reviews of Interventions (25), which lists seven items: random sequence generation, allocation concealment, blinding of participants and personnel, blinding of outcome assessment, incomplete outcome data, selective reporting, and other forms of bias.

The scientific quality of the included studies was assessed by two independent authors (QQW and HTH). Differences related to the judgment of the quality of evidence included in each study were resolved through discussion and consensus with a third author (DXH).

### Data Analysis

Data analysis was carried out with Review Manager Version 5.4 software. We determined risk ratios (RRs) and 95% confidence intervals (CIs) for categorical variables. Continuous data were presented as mean difference (MD) and its 95% CIs. We also used a random effect miniature to aggregate research studies that showed serious heterogeneity, determined the inconsistency index (*I*^2^ ≥ 30%), and used a fixed effect miniature to merge studies in case of significant heterogeneity (*I*^2^ <30%). We also carried out a sensitivity analysis to identify the influence of confounding parameters. Funnel plot was used to evaluate publication bias; statistical significance was set at *p* < 0.05 (26).

## Results

### Trial Identification

**Figure 1:**
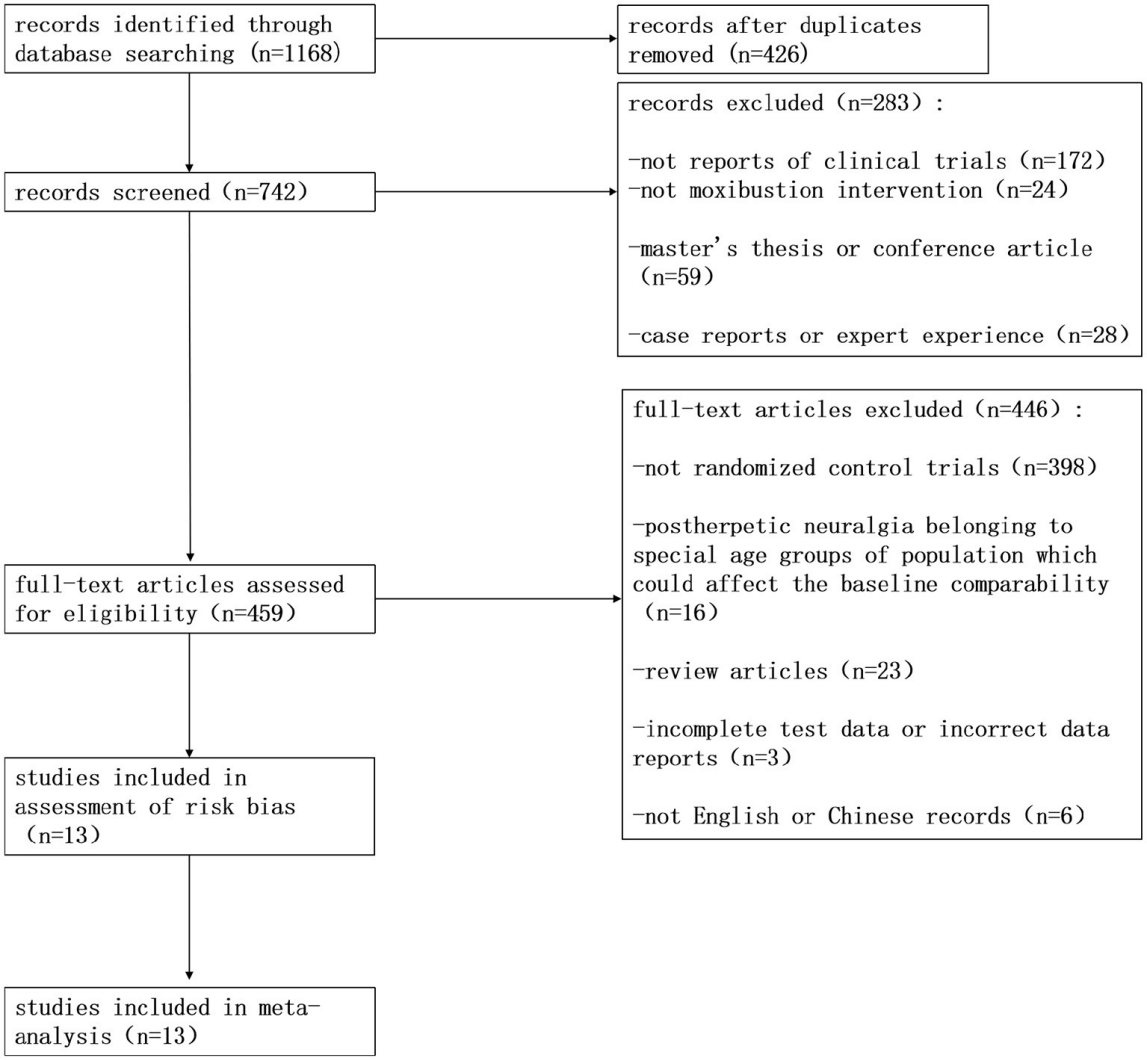
Flowchart of the process used to retrieve relevant articles from the literature.

Our literature searches identified 1,168 relevant records. We screened titles and abstracts and considered that 459 RCTs could potentially be included. After analyzing the entire collection of studies, we excluded 446 RCTs. Therefore, our analysis included 13 studies (Figure 1).

### Characteristics of the Included Studies

The quality of the 13 included RCTs (*n* = 798; moxibustion: *n* = 402, control: *n* = 396) are summarized in Table 1. Five studies (27–31) involved Zhuang medicine medicated thread moxibustion, one trial (32) involved juncus moxibustion, one study (33) involved grain-moxibustion, two trials (34, 35) involved thunder-fire moxibustion, one trial (36) involved heat-sensitive moxibustion, one study (37) involved sky moxibustion, one trial (38) involved electronic moxibustion, and one trial (39) involved Mongolian moxibustion.

**Table 1:** The characteristics of the randomized controlled trials (RCTs) included in this meta-analysis.

**Included trials**	**Eligibility criteria**	**Intervention and treatment**		**Sample and characteristics (male/female, age, disease duration)**		**Outcomes**
		**Trial**	**Control**	**Trial**	**Control**	
Bai et al. (27)	TAAN	Light Zhuang medicine medicated thread moxibustion Duration: 30 days	Pharmacological (indomethacin, dipyridamole, carbamazepine) Duration: 30 d	20 (M:5, F:15); AGE: (mean: 71.22 ± 5.63 years); Disease duration: (mean:6.23 ± 2.21 months)	20 (M:6, F:14); AGE: (mean: 72.54 ± 3.67 years); Disease duration: (mean: 6.19 ± 2.11 months)	Clinical efficacy rate VAS Adverse reactions
Li et al. (28)	Unclear	Zhuang medicine medicated thread moxibustion Duration: 28 days	Pharmacological (ibuprofen, cimetidine) Duration: 28 d	29 (M:-, F:-); AGE: 34–78; Disease duration: 3.5 months−2 years	27 (M:-, F:-); AGE: 41–82; Disease duration: 4 months−1.9 years	Clinical efficacy rate VAS Adverse reactions
Lin et al. (29)	ANA	Zhuang medicine medicated thread moxibustion Duration: 28 days	Pharmacological (ibuprofen, cimetidine) Duration: 28 d	30 (M:17, F:13); AGE: (mean: 62.79 ± 3.69 years); Disease duration: (mean:5.9 ± 1.44 months)	30 (M:14, F:16); AGE: (mean: 62.51 ± 3.87 years); Disease duration: (mean:6.07 ± 1.27 months)	Clinical efficacy rate VAS Adverse reactions
Zhong et al. (30)	DV-1	Zhuang medicine medicated thread moxibustion Duration: 14 days	Pharmacological (recombinant human interferonα2a for injection, vitamin B12) Duration: 14 d	28 (M:16, F:12); AGE: 31–72; Disease duration:-	26 (M:17, F:9); AGE: 30–74; Disease duration:-	Clinical efficacy rate Adverse reactions
Fang et al. (31)	TAAN	Zhuang medicine medicated thread moxibustion Duration: 30 days	No treatment Duration: 30 d	20 (M:-, F:-); AGE:-; Disease duration:-	20 (M:-, F:-); AGE:-; Disease duration:-	VAS
Lin et al. (32)	DV-7	Juncus moxibustion Duration:-d	Pharmacological (acyclovir, prednisone, indomethacin) Duration:-d Herbal Duration:-d	20 (M:-, F:-); AGE: 28–83; Disease duration:2 months−2 years	(1) 19 (M:-, F:-); AGE: 28–83; Disease duration: 2 months−2 years (2) 20 (M:-, F:-); AGE: 28–83; Disease duration: 2 months−2 years	Clinical efficacy rate
Zhou et al. (33)	CECDTPHN CDTETCMDS	Grain-moxibustion Duration: 35 days	Pharmacological (vitamin B1, mecobalamin, gabapentin) Duration: 35 d	32 (M:17, F:15); AGE: (mean: 54.13 ± 12.85 years); Disease duration: (mean: 101.56 ± 55.17 d)	32 (M:14, F:18); AGE: (mean: 52.5 ± 13.42 years); Disease duration: (mean: 102.16 ± 53.46 d)	Clinical efficacy rate VAS
Wang et al. (34)	CDTETCMDS	Thunder-fire moxibustion +pharmacological Duration: 21 days	Pharmacological (gabapentin) Duration: 21 d	30 (M:16, F:14); AGE: 44–75; Disease duration: 1–18 months	30 (M:13, F:17); AGE: 46–77; Disease duration: 1–15 months	Clinical efficacy rate
Ye et al. (35)	Modern Dermatology	Thunder-fire moxibustion +pharmacological Duration:-d	Pharmacological (meloxicam, mecobalamin) Duration:-d	30 (M:17, F:13); AGE: (mean: 46.82 ± 7.56 years); Disease duration:-	30 (M:15, F:15); AGE: (mean: 47.92 ± 8.31 years); Disease duration:-	Clinical efficacy rateVAS
Cao et al. (36)	Clinical Diagnosis and Treatment Guide (Pain Volume) GCTNPCM	Heat-sensitive moxibustion +pharmacological Duration: 28 days	Herbal+pharmacological (valacyclovir hydrochloride, vitamin B1, indomethacin) Duration: 28 days	43 (M:16, F:27); AGE: 30–67 years; Disease duration: 47 days−5 months	43 (M:17, F:26); AGE: 28–65 years; Disease duration: 42 days−4 months	Clinical efficacy rate VAS
Jin et al. (37)	CDTETCMDS	Sky moxibustion Duration: 21 days	Herbal Duration: 21 days	45 (M:-, F:-); AGE: 30–81 years; Disease duration:-	44 (M:-, F:-); AGE: 30–81 years; Disease duration:-	Clinical efficacy rate
Wu et al. (38)	Unclear	Electronic moxibustion Duration: 23 days Electronic moxibustion + pharmacological Duration: 23 days	Pharmacological (gabapentin) Duration: 30 days	(1) 20 (M:12, F:8); AGE: 30–68 years; Disease duration: 3 months−1 years(2) 20 (M:10, F:10); AGE: 33–65 years; Disease duration: 3 months−1 years	20 (M:11, F:9); AGE: 31–69 years; Disease duration: 2 months−1 years	Clinical efficacy rate VAS Adverse reactions
Zhou et al. (39)	DV-7	Mongolian moxibustion Duration: 14 days	Pharmacological (acyclovir) Duration: 14 days	35 (M:-, F:-); AGE: 18–85 years; Disease duration: 3–60 months	35 (M:-, F:-); AGE: 18–85 years; Disease duration: 3–60 months	Clinical efficacy rate VAS

*TAAN, The American Academy of Neurology; VAS, Visual Analog Scale; ANA, American Neurological Association; DV-1, Dermatology and Venerology first edition; DV-7, Dermatology and Venerology 7th edition; CECDTPHN, Chinese expert consensus on diagnosis and treatment of postherpetic neuralgia; CDTETCMDS, Criteria of Diagnosis and Therapeutic Effects for TCM Disease and Syndrome; GCTNPCM, Guideline for Clinical Trials of New Patent Chinese Medicine*.

The age range of the patients featured in the 13 trials was 18–85 years (39), and the total duration of disease was (34) 1 to 60 months (39).

The diagnostic criteria for the 13 trials were as follows: (1) The American Academy of Neurology (TAAN) (27, 31); (2) The American Neurological Association (ANA) (29); (3) Dermatology and Venerology First Edition (DV-1) (30); (4) Dermatology and Venerology Seventh edition (DV-7) (32, 39); (5) The Chinese Expert Consensus on Diagnosis and Treatment of Postherpetic Neuralgia (CECDTPHN) (33); (6) The Criteria of Diagnosis and Therapeutic Effects for TCM Disease and Syndrome (CDTETCMDS) (33, 34, 37); (7) Modern Dermatology (35); (8) Clinical Diagnosis and Treatment Guide (Pain Volume) (36); (9) Guideline for Clinical Trials of New Patent Chinese Medicine (GCTNPCM) (36); and (10) unclear criteria (28, 38).

The duration of moxibustion treatment described in the 13 RCTs ranged from 14 days (30, 39) to 35 days (33).

Regarding the types of the control group, nine studies (27–30, 33–35, 38, 39) adopted pharmacological therapies as the control; three studies (32, 36, 37) used herbal medicine as the control; and one trial did not involve a specific treatment (31). Pharmacological therapies included indomethacin, dipyridamole, carbamazepine, ibuprofen, cimetidine, recombinant human interferonα2a for injection, acyclovir, prednisone, vitamin B1, mecobalamin, gabapentin, meloxicam, and valacyclovir hydrochloride. The duration of treatment in the control group ranged from 14 days (30, 39) to 35 days (33).

The definition of efficacy rate was classified according to five criteria: (1) CDTETCMDS (32, 34), (2) Clinical disease diagnosis based on improvement criteria (33), (3) Modern Chinese Medicine Dermatology (36), (4) Diagnosis and curative effect criteria of Mongolian medicine (39), and (5) unclear criteria (27, 30). All these criteria for efficacy rate listed in the included studies were mainly based on change of pain (VAS), except for two studies (28, 35), which stated that “clinical efficacy rate” was calculated based on change of either “VAS” or Sleep Quality Scale (SQS).

The baseline characteristics of the included studies were similar as there were no significant differences between the intervention group and control group with regards to disease duration, age, or gender (*P* > 0.05).

### Risk of Bias in the Included Studies

**Figure 2:**
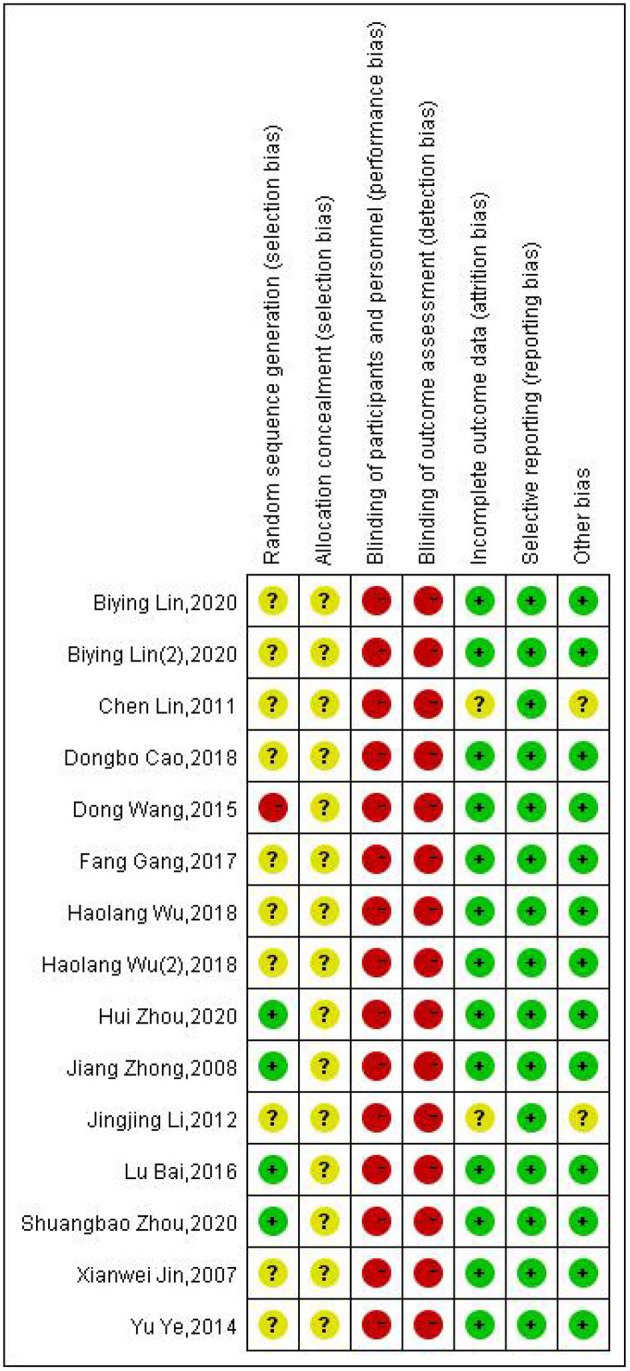
An evaluation of risk bias in the included randomized controlled trials (RCTs).

The risk of bias in the 13 included RCTs was generally high (Figure 2).

In one RCT (34), the risk of bias regarding random sequence generation was high due to using inappropriate randomization methods. In four trials (27, 30, 33, 39), random sequence generation showed that the risk of bias was low owing to the use of a random number table. In eight trials (28, 29, 31, 32, 35–38), we found that the risk of bias was unclear owing to the lack of sufficient detail in the articles.

In all 13 studies (27–39), the risk of bias as arising from allocation concealment was unclear owing to the lack of sufficient detail in the articles.

In all 13 articles (27–39), we found that the risk of bias owing to the blinding of the participants was high. The impossibility to blind the participants was because moxibustion (a treatment of procedural nature) was compared to pharmacological and/or herbal therapies.

In all 13 trials (27–39), the risk of bias due to the blinding of outcome assessments was high because the outcome assessors that interviewed the subjects about the outcomes were not blinded to their allocation between the trial's arms.

In 11 RCTs (27, 30–39), the risk of attrition bias was low. This was because there were no missing data as the authors recorded all of the expected results. The risk of bias associated with two of the RCTs (28, 29) was unclear because they reported insufficient details to ensure that the baseline was balanced after dropping out.

In all 13 studies (27–39), we found that the risk of bias owing to selective reporting was low as all RCTs recorded all of the expected data required by their study protocol.

In 11 tests (27, 30–39), we found that the risk of bias due to other reasons was low because these studies did not appear to have other sources of bias. Two articles (28, 29) were classified as having an unclear risk because there were not enough details to ensure that the baseline was balanced after patients dropped out of the trials.

### The Clinical Efficacy Rate of Moxibustion for the Treatment of PHN

**Figure 3:**
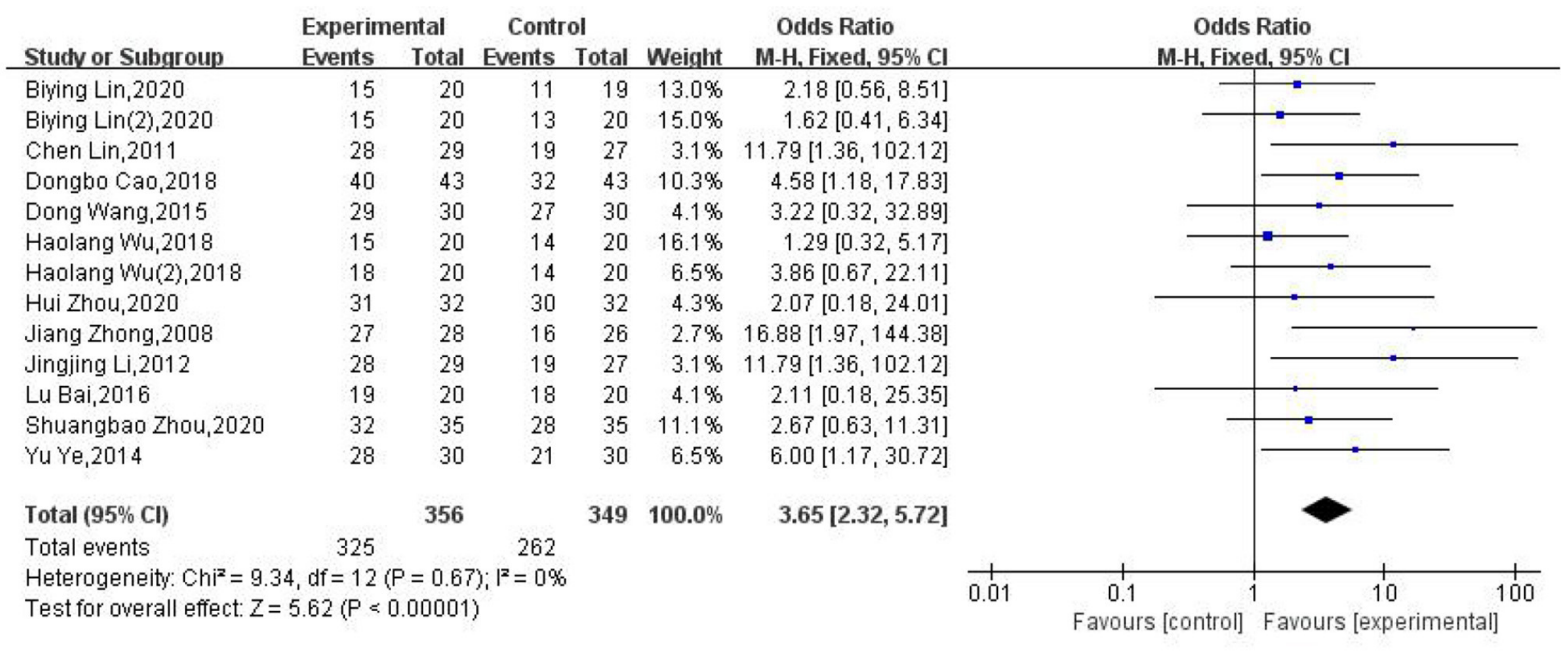
Forest plot and meta-analysis of the clinical efficacy rate of moxibustion in the treatment of PHN.

We analyzed the clinical efficacy rate for moxibustion in the treatment of PHN in 11 of the included RCTs. Our meta-analysis showed that moxibustion was superior to pharmacological therapies, herbal medicine, and no treatment (OR: 3.65; 95% CI: [2.32, 5.72]; *P* < 0.00001, Figure 3). There was no evidence of serious heterogeneity between the tests (χ^2^ = 9.34; *P* = 0.67; *I*^2^ = 0%).

Because the interventions used in different articles were inconsistent, we analyzed the control group interventions, treatment processes, and moxibustion methods, as subgroups. Irrespective of the subgroup analysis, the conclusions remained consistent.

### Control Group Intervention

**Figure 4:**
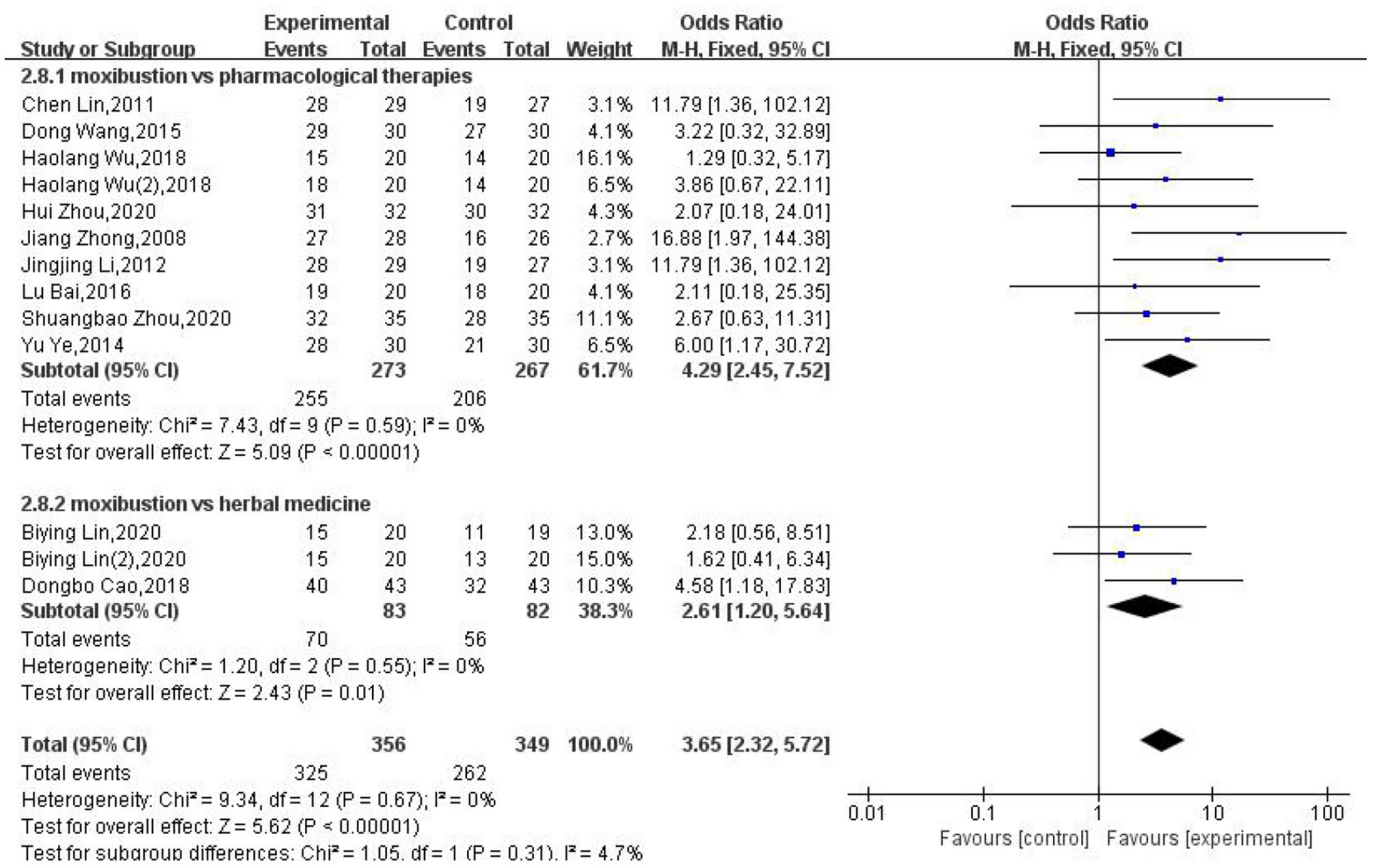
Subgroup analysis of different interventions in the control groups.

In terms of the types of controls, the interventions could be classified as pharmacological therapies and herbal medicine. Analysis showed that moxibustion had a better clinical effect (OR: 4.29; 95% CI: [2.45, 7.52]; *P* < 0.00001; OR: 2.61; 95% CI: [1.2, 5.64]; *P* = 0.01) in relation to pharmacological therapies and herbal medicine, respectively (Figure 4).

### Treatment Course

**Figure 5:**
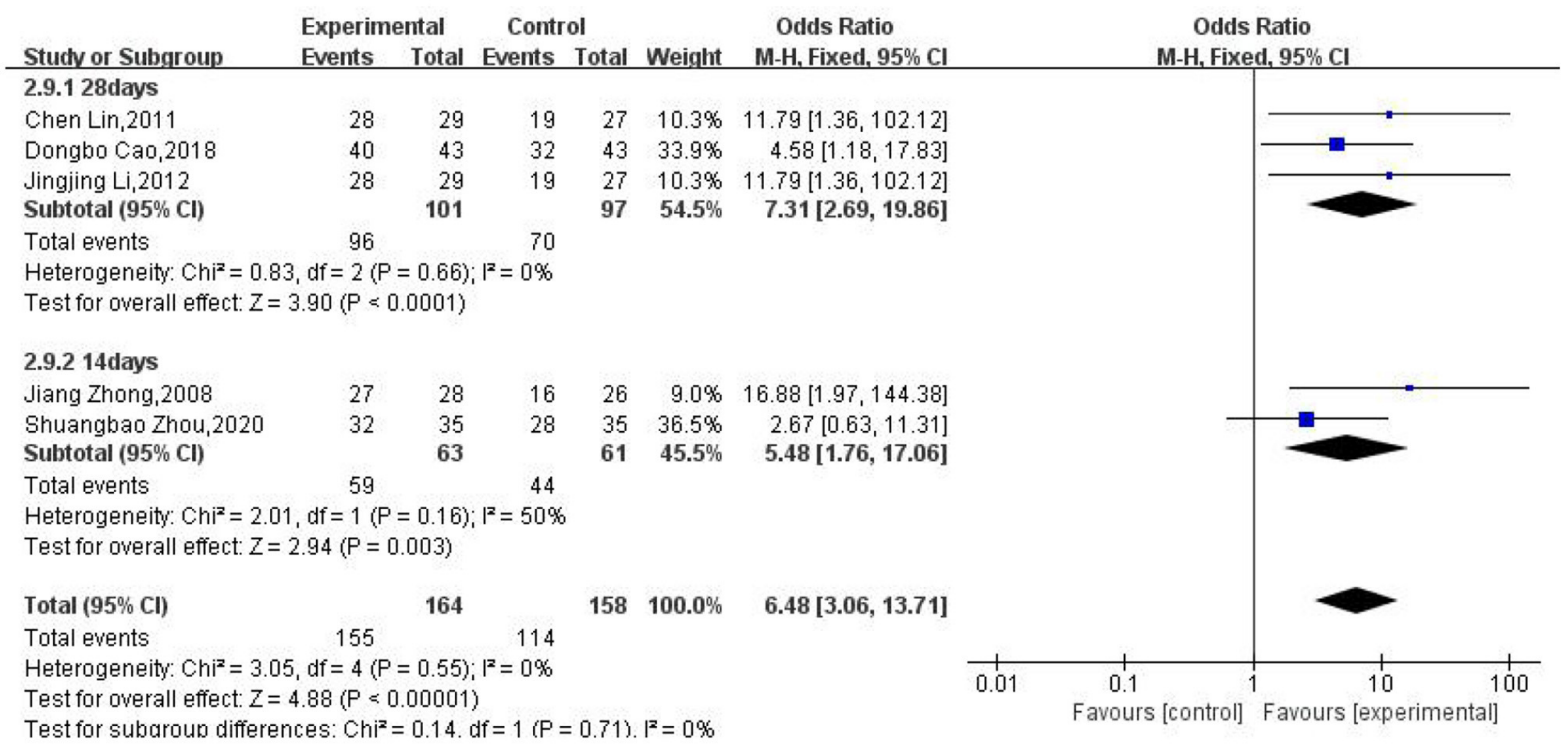
Subgroup analysis of different treatment courses.

To identify the effect of different treatment courses on the clinical efficacy rate, we performed subgroup analysis in terms of the different treatment courses. We found that the clinical efficacy of moxibustion over 28 days (OR: 7.31; 95% CI: [2.69, 19.86]; *P* < 0.0001) was higher than that over 14 days (OR: 5.48; 95% CI: [1.76, 17.06]; *P* = 0.0003; Figure 5).

### The Modalities of Moxibustion

**Figure 6:**
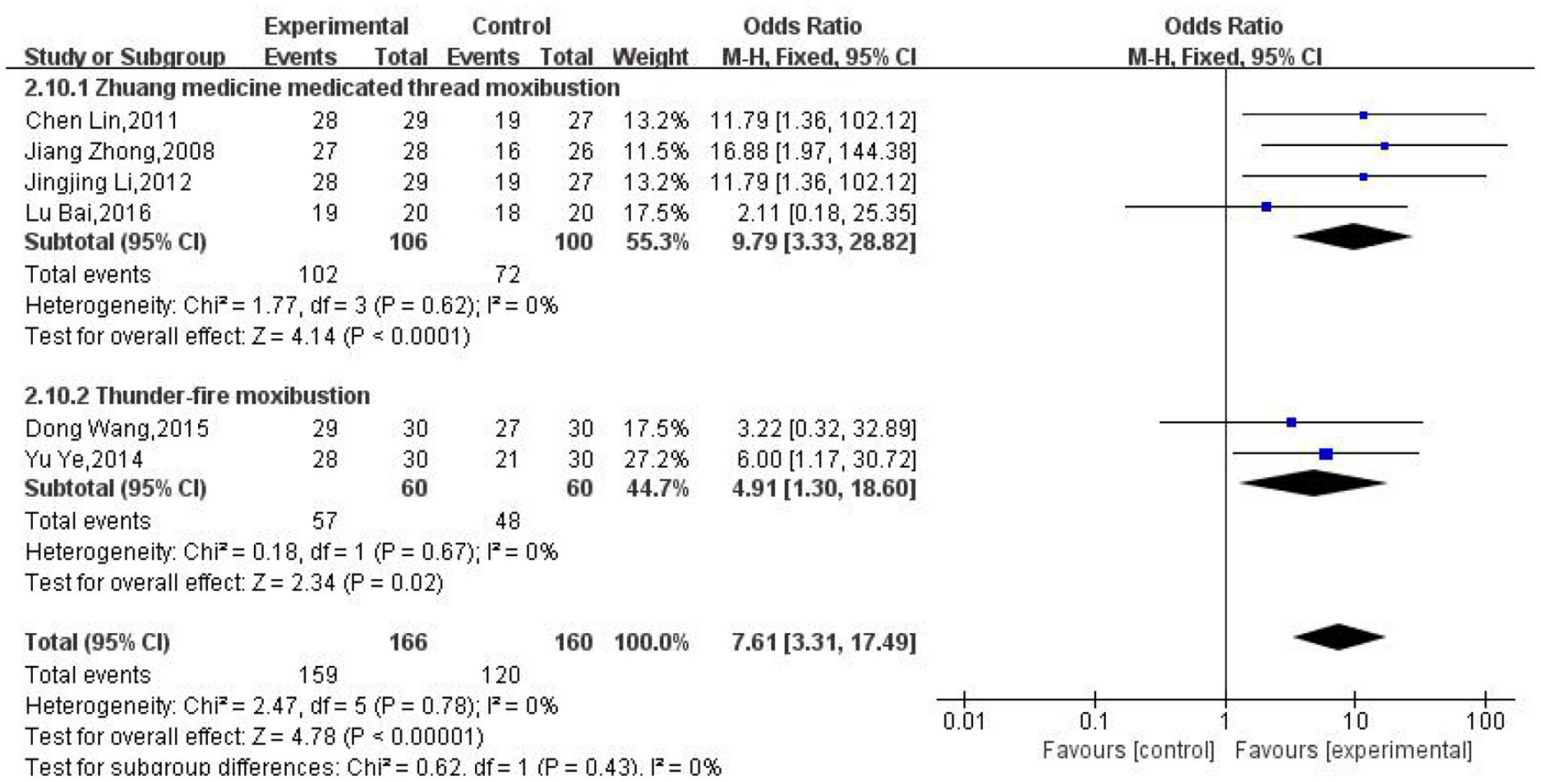
Subgroup analysis of the different modalities used for moxibustion treatment.

We also conducted subgroup analysis based on various moxibustion modalities, which found that the clinical efficacy rate of Zhuang medicine medicated thread moxibustion and thunder-fire moxibustion was higher than that of the control group (OR: 9.79; 95% CI: [3.33, 28.82]; *P* < 0.0001; OR: 4.91; 95% CI: [1.3, 18.6]; *P* = 0.02, respectively, Figure 6).

### VAS

**Figure 7:**
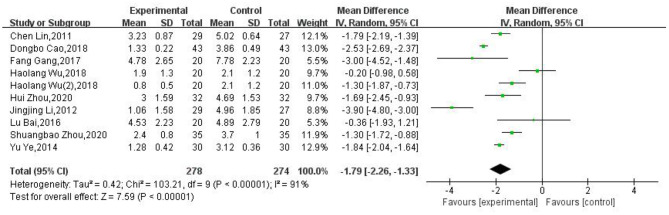
Meta-analysis of VAS data.

Nine studies (27–29, 31, 33, 35, 36, 38, 39) used the VAS to assess the pain score (in 552 patients). The *X*^2^ test indicated substantial heterogeneity among these studies (*P* < 0.00001, *I*^2^ = 91%). Therefore, we used random-effects models (REM) for analysis. The moxibustion group was associated with a lower VAS than the control group. Significant differences were observed between groups (MD: −1.79; 95% CI: [−2.26, −1.33]; *P* < 0.00001, Figure 7). Nevertheless, given the relatively small size of the effect, it is questionable whether this difference is clinically relevant.

### Adverse Reactions

**Figure 8:**
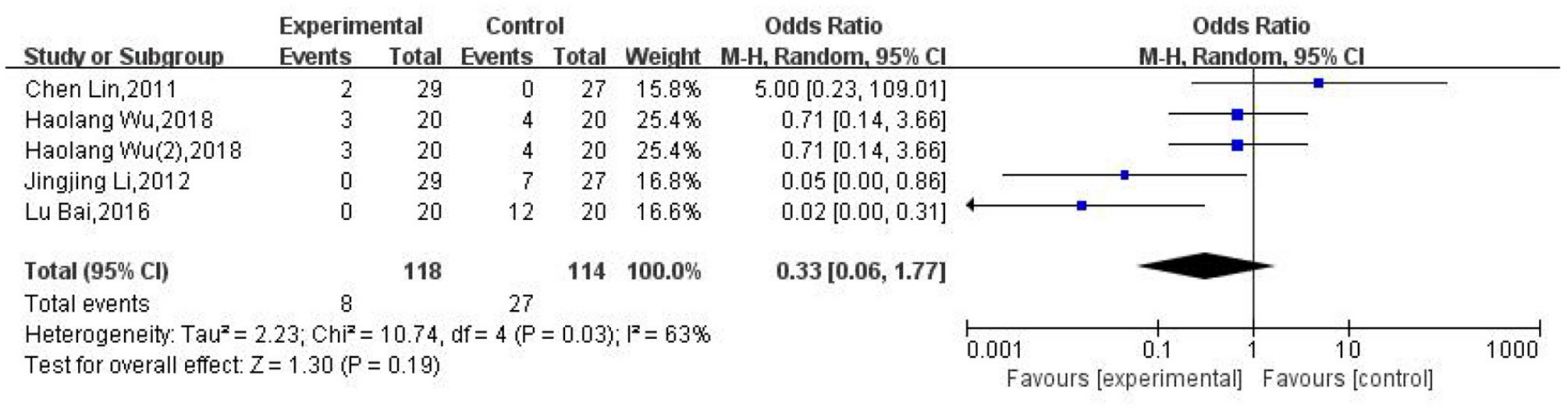
Meta-analysis of adverse events.

Of the 13 RCTs included in our analysis, five (27–30, 38) reported adverse reactions after moxibustion; no significant adverse reactions were reported in one report (30). Four studies (27–29, 38) reported adverse reactions, as showed in Figure 8. The study by Wu (38) reported two cases (10%) of dizziness, one case (5%) of nausea and vomiting associated with electronic moxibustion, one case (5%) of abdominal distention, and two cases (10%) of nausea and vomiting (a total of 3 cases, 15%) in a group treated with a combination of electronic moxibustion and gabapentin. In the gabapentin group, there was one case (5%) of dizziness, one case (5%) of distention, and two cases (10%) of nausea and vomiting (a total of 4 cases, 20%). Another study (30), reported seven cases (25.9%) of nausea, vomiting, and abdominal discomfort in a group treated with fenbid and cimetidine tablets. Lin et al. (29) reported two cases (6.9%) of Zhuang medicine-medicated thread moxibustion burns. Bai et al. (27) reported redness and itching in five cases (25%) and three cases of blisters and infection (15%) in the Zhuang medicine-medicated thread moxibustion group along with gastrointestinal adverse reactions in seven cases (35%), three cases (15%) of rash, and two cases (10%) of mild liver dysfunction in the group treated with pharmacological therapies. These results showed that moxibustion was as safe (OR: 0.33; 95% CI: [0.06, 1.77]; *P* = 0.19; Figure 8) as pharmacological therapies.

### Publication Bias

**Figure 9:**
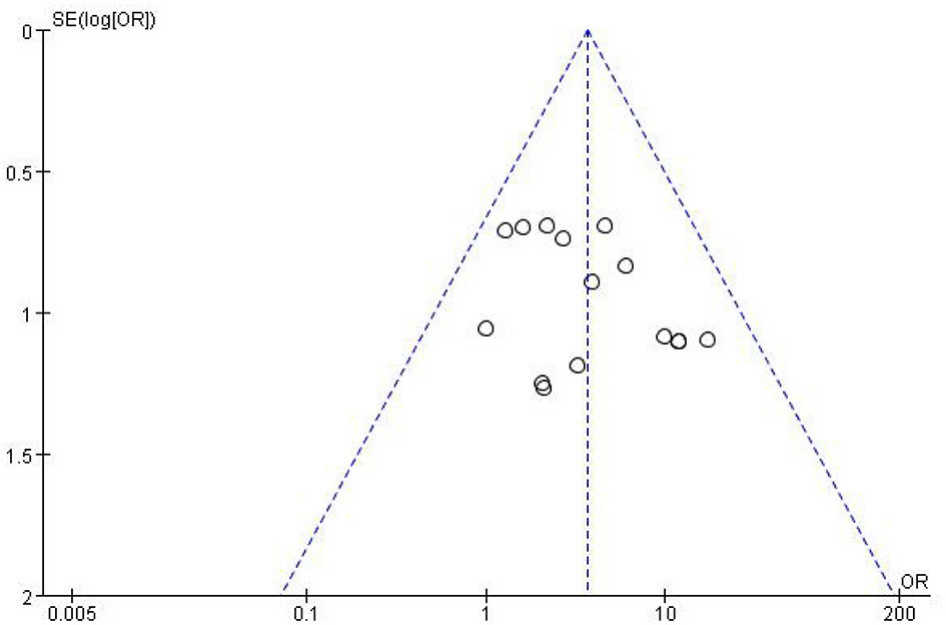
Funnel plot of publication bias.

Our analyses identified publication bias, the inverted funnel diagram shows the poor symmetry on both sides and significantly to the left, suggesting the publication bias, as shown in Figure 9.

## Discussion

### Summary of Results

Our analysis showed that the analgesic effect of moxibustion on PHN was superior to that of pharmacological therapies, herbal medicine, and no treatment both in the overall meta-analysis and subgroup meta-analysis. Nonetheless, the efficacy of moxibustion in the treatment of PHN remains uncertain because of the inferior methodological quality and the significant heterogeneity of the included studies. In addition, only a few studies have reported the safety of moxibustion; therefore, it was not possible for our meta-analysis to determine the safety of moxibustion for treating PHN.

### Quality of the Evidence

Our findings should be considered with caution because of the high risk of bias in the RCTs included in our analysis. In particular, it is necessary to consider the risk of bias owing to random sequence generation, concealment allocation, the blinding of participants and personal information, the blinding of outcome assessments, and incomplete outcome data. First, random sequence generation and concealment allocation are crucial if we are to preclude option bias. For example, it is reported that inadequate or unclear allocation concealment in trials could lead to overestimation of the benefit of an intervention in an average of 18% (95% CI 5–29%) when compared to effect estimates from trials with adequate concealment (40). Second, the blinding of participants and outcome assessments are very important for bias avoidance. However, the risk of bias regarding these two items is high because blinding was not conducted.

### Limitations

There are some limitations to our meta-analysis that need to be addressed. First, there was significant heterogeneity among the included studies regarding the interventions, both for the experimental and control groups. There was great variability in the modality of moxibustion applied. As the efficacy of each of these modalities may differ significantly, the results for the pooled analysis of the trials may not be generalizable for all the moxibustion modalities that were included. Second, as moxibustion applied in the experimental group is a procedural intervention and the control group did not involve a similar procedural intervention, it is possible that the differences observed between the pooled experimental and control groups may be at least partially justified by differences in placebo effect of these interventions. It should particularly be noted given that none of the included trials conducted blinding for outcome assessment. Third, this meta-analysis selected clinical efficacy rate as a primary endpoint, because it is a very frequently reported outcome in Chinese RCTs, while it is not widely used and acknowledged. This may significantly limit the comparability of the results obtained from this meta-analysis with those of other trials and meta-analyses of the literature. Additionally, the standard for calculating the efficacy rate varied significantly among different trials, thereby greatly reducing the validity and reliability of the efficacy rate (41). Thus, it is worth noting that the meta-analysis result of efficacy rate should be interpreted with extreme caution. Fourth, the publication bias observed in our meta-analysis should be noted given that all included studies were conducted in China. It is reported that there is a strong publication bias for acupuncture articles conducted in China, where the percentage of positive results of RCTs is fairly high when compared to Western acupuncture RCTs. The exclusion of master's thesis and conference articles in our meta-analysis may also lead to an increased risk of publication bias. Fifth, the lack of methodological quality among the included studies also limits the robustness of the results of this meta-analysis.

Last but not least, it is notable that in seven of the nine included trials that compared moxibustion to pharmacological therapies, no well-established medication for neuropathic pain treatment was used. In addition, the three trials (33, 34, 38) that adopted gabapentin as a control only had treatment durations of 21, 30, and 35 days. These small time periods may not be enough for the efficacy of this medication to be fully assessed. Thus, in further studies that aim to compare moxibustion to pharmacological treatments, more attention should be paid to the selection of medications with well-established efficacy for neuropathic pain and longer treatment duration.

### Implications

Our meta-analysis shows that more high-quality RCTs are necessary before drawing a firm conclusion regarding the efficacy and safety of moxibustion for treating PHN. When designing and implementing prospective moxibustion RCTs for PHN, we suggest that researchers follow the CONSORT 2010 statement (42, 43). This statement consists of a list of 25 items to determine the quality and rigor of a trial, which should be used as a standard guideline. According to the International Committee of Medical Journal Editors statement (44), all clinical trials should be registered before the first patient is enrolled. Furthermore, the randomization method should be clearly described, and the report should be adequately detailed. Although blinding can be difficult, it is important that we attempt to blind patients and outcome assessments. Clear and widely accepted diagnostic or classification criteria (e.g., the CDTETCMDS, TAAN, or DV-7) should be used for the accurate diagnosis of PHN; this should improve the comparability between different RCTs. Intervention details regarding treatment courses, treatment frequency, and duration of follow-up should be reported adequately. Irrespective of treatment, the severity of PHN may vary; therefore, a longer follow-up time for the continuous measurement of results is crucial to assess the long-term outcomes of moxibustion. Measurements that are internationally acknowledged and formalized should be consistently combined and applied. It should be noted that none of the included RCTs in our meta-analysis provided outcome measurements in terms of quality of life, functionality, and mood. Similarly, outcome measurements of sleep quality (e.g., SQS) were also rarely reported in the included trials. Although two trials (28, 35) stated that one of their reported outcomes “efficacy rate” was calculated based on change of either VAS or SQS, the original data regarding SQS were not provided in their studies. Therefore, future RCTs in this field should pay extra attention to include such outcome measurements. In addition, it is also vital to include a sufficiently large sample size to ensure statistical power is retained.

## Conclusions

Due to the lack of methodological quality as well as the significant heterogeneity of the included studies, it remains difficult to draw a firm conclusion on the efficacy and safety of moxibustion for the treatment of PHN. Future high-quality studies are urgently needed.

## Data Availability Statement

The original contributions presented in the study are included in the article/Supplementary Material, further inquiries can be directed to the corresponding authors.

## Author Contributions

QW: concept and design. QW, HH, DH, and HG: data acquisition and amendment of the manuscript. QW and HH: data analysis and interpretation. QW: drafting of manuscripts. All authors agreed with the concluding edition of the manuscript.

## Conflict of Interest

The authors declare that the research was conducted in the absence of any commercial or financial relationships that could be construed as a potential conflict of interest.

## Publisher's Note

All claims expressed in this article are solely those of the authors and do not necessarily represent those of their affiliated organizations, or those of the publisher, the editors and the reviewers. Any product that may be evaluated in this article, or claim that may be made by its manufacturer, is not guaranteed or endorsed by the publisher.

## Supplementary Material

Appendix S1 Prisma checklist.

Appendix S2 Search strategy.
