# Supplementary material for: Efficacy and Safety of Moxibustion for Postherpetic Neuralgia: A Systematic Review and Meta-Analysis
Source: Front Neurol. 2021 Aug 26;12:676525. doi: 10.3389/fneur.2021.676525 (PMC8427698; doi:10.3389/fneur.2021.676525)
Supplement: Appendix S2 — Search strategy. [file Data_Sheet_2.doc]

**1. PUBMED (Search date: January 13, 2021)**

Search terms ：("moxibustion"[MeSH Terms] OR "moxibustion"[All Fields] OR "moxa"[All Fields]) AND ("herpes zoster"[MeSH Terms] OR "herpes zoster"[All Fields] OR "neuralgia, postherpetic "[MeSH Terms] OR "postherpetic neuralgia"[All Fields] OR "zona"[All Fields] OR "zoster"[All Fields] OR "shingles"[All Fields] AND (("random allocation"[MeSH Terms] OR ("random"[All Fields] AND "allocation"[All Fields]) OR "random allocation"[All Fields] OR "randomized"[All Fields]) OR ("clinical trials as topic"[MeSH Terms] OR ("clinical"[All Fields] AND "trials"[All Fields] AND "topic"[All Fields]) OR "clinical trials as topic"[All Fields] OR "trial"[All Fields]))

**2. EMBASE (Search date: January 13, 2021)**

Search terms ：'moxibustion'/exp OR moxibustion OR 'moxa'/exp OR moxa AND ('herpes zoster'/exp OR herpes zoster OR 'postherpetic neuralgia'/exp OR postherpetic neuralgia OR 'zona'/exp OR zona OR 'zoster'/exp OR zoster OR 'shingles'/exp OR shingles) AND (randomized OR trial)

**3. The Cochrane Library (Search date: January 13, 2021)**

Search terms ：1. herpes zoster*:ti,ab OR postherpetic neuralgia*:ti,ab OR zona*:ti,ab OR zoster*:ti,ab OR shingles*:ti,ab OR zoster herpes*:ti,ab

2. MeSH descriptor: [Herpes Zoster] explode all trees

3. MeSH descriptor: [Neuralgia, Postherpetic] explode all trees

4. #1 OR #2 OR #3

5. MeSH descriptor: [Moxibustion] explode all trees

6. moxibustion:ti,ab OR moxa:ti,ab

7. #5 OR #6

8. #4 AND #7 (limited to “trials”)

**4. International Clinical Trials Register Platform of WHO (Search date: January 13, 2021)**

Search terms：moxibustion AND postherpetic neuralgia

**5. Web of science (Search date: January 13, 2021)**

Search terms: (moxibustion*[Title/Abstract] OR moxibustion*[TOPIC] OR moxabustion*[TOPIC] OR moxa*[TOPIC]) AND (herpes zoster*[Title/Abstract] OR postherpetic neuralgia*[Title/Abstract] OR herpes zoster*[TOPIC] OR postherpetic neuralgia*[TOPIC] OR zona*[TOPIC] OR zoster*[TOPIC] OR shingles*[TOPIC] OR zoster herpes*[TOPIC])

**6. WanfangData (Search date: January 13, 2021)**

Search terms:主题:灸*("带状疱疹"+"蛇串疮"+"缠腰火丹"+"火带疮"+"火丹"+"带状性疱疹"+"蜘蛛疮"+"蛇盘疮"+"蛇丹"+"带状疱疹后遗神经痛")

**7. China National Knowledge Infrastructure (CNKI) (Search date: January 13, 2021)**

Search terms: TI=灸*(带状疱疹+蛇串疮+缠腰火丹+火带疮+火丹+带状性疱疹+蜘蛛疮+蛇盘疮+蛇丹+带状疱疹后遗神经痛) AND AB=灸*(带状疱疹+蛇串疮+缠腰火丹+火带疮+火丹+带状性疱疹+蜘蛛疮+蛇盘疮+蛇丹+带状疱疹后遗神经痛)

**8. VIP Database for Chinese Technical Periodicals (VIP) (Search date: January 13, 2021)**

Search terms: U=灸 AND (U=带状疱疹 OR U=蛇串疮 OR U=缠腰火丹 OR U=火带疮OR U=火丹 OR U=带状性疱疹 OR U=蜘蛛疮 OR U=蛇盘疮 OR U=蛇丹 OR U=带状疱疹后遗神经痛)

1. **China Biomedical Network Information (Search date: January 13, 2021)**

Search terms: (灸法[主题词] OR 灸[全部字段]) AND (带状疱疹[主题词] OR 带状疱疹后遗神经痛[主题词] OR 带状疱疹[全部字段] OR 带状疱疹后遗神经痛[全部字段] OR蛇串疮[全部字段] OR 缠腰火丹[全部字段] OR 火带疮[全部字段] OR 火丹[全部字段] OR 带状性疱疹[全部字段] OR 蜘蛛疮[全部字段] OR 蛇盘疮[全部字段] OR 蛇丹[全部字段] OR 带状疱疹后遗神经痛[全部字段])
